# Comparison of the diagnostic accuracy of resting-state fMRI driven machine learning algorithms in the detection of mild cognitive impairment

**DOI:** 10.1038/s41598-023-49461-y

**Published:** 2023-12-14

**Authors:** Gergo Bolla, Dalida Borbala Berente, Anita Andrássy, Janos Andras Zsuffa, Zoltan Hidasi, Eva Csibri, Gabor Csukly, Anita Kamondi, Mate Kiss, Andras Attila Horvath

**Affiliations:** 1 Neurocognitive Research Center, National Institute of Mental Health, Neurology and Neurosurgery, Budapest, Hungary; 2 School of PhD Studies, Semmelweis University, Budapest, Hungary; 3 Department of Family Medicine, Semmelweis University, Budapest, Hungary; 4 Department of Psychiatry and Psychotherapy, Semmelweis University, Budapest, Hungary; 5 Department of Neurology, Semmelweis University, Budapest, Hungary; 6 Siemens Healthcare, Budapest, Hungary; 7 Department of Anatomy Histology and Embryology, Semmelweis University, Budapest, Hungary

**Keywords:** Neurological disorders, Biomarkers

## Abstract

Mild cognitive impairment (MCI) is a potential therapeutic window in the prevention of dementia; however, automated detection of early cognitive deterioration is an unresolved issue. The aim of our study was to compare various classification approaches to differentiate MCI patients from healthy controls, based on rs-fMRI data, using machine learning (ML) algorithms. Own dataset (from two centers) and ADNI database were used during the analysis. Three fMRI parameters were applied in five feature selection algorithms: local correlation, intrinsic connectivity, and fractional amplitude of low frequency fluctuations. Support vector machine (SVM) and random forest (RF) methods were applied for classification. We achieved a relatively wide range of 78–87% accuracy for the various feature selection methods with SVM combining the three rs-fMRI parameters. In the ADNI datasets case we can also see even 90% accuracy scores. RF provided a more harmonized result among the feature selection algorithms in both datasets with 80–84% accuracy for our local and 74–82% for the ADNI database. Despite some lower performance metrics of some algorithms, most of the results were positive and could be seen in two unrelated datasets which increase the validity of our methods. Our results highlight the potential of ML-based fMRI applications for automated diagnostic techniques to recognize MCI patients.

## Introduction

Alzheimer’s disease (AD) is the most frequent type of major neurocognitive disorders leading to progressive deterioration of cognitive abilities^1^. AD is a growing health problem worldwide, and the major scientific challenge is to diagnose the related pathology before symptoms manifest^2^. Detecting and addressing mild cognitive impairment (MCI) is an important step to slow down the progression of cognitive decline^3^. Biomarkers identifying individuals who are at higher risk for developing dementia are necessary, allowing early intervention^4^. Various biomarkers such as genetics, lab tests, neurophysiology, and medical imaging were examined and compared regarding their diagnostic and prognostic value. Nevertheless, today there is no method with adequate efficiency for diagnosing MCI without the application of a combined biomarker setup^5^. Positron emission tomography (PET) and cerebrospinal fluid (CSF) analysis can reach the highest individual efficacy; however, both methods have drawbacks (CSF is invasive, PET scans are expensive and utilize ionizing radiation). Magnetic resonance imaging (MRI) is a non-invasive approach providing several options to detect the disease-related changes. Structural MRI (sMRI) depicts brain atrophy, diffusion tensor imaging (DTI) describes white matter integrity, functional MRI (fMRI) gives a glimpse about the patient’s actual brain activation. Furthermore, early studies already showed that low frequency fMRI fluctuations can be used to determine the functional connectivity of large-scale neural networks^6^. The absence, strengthening or weakening of these networks may indicate the presence of MCI and AD pathology^7^. Regarding the latest research, fMRI shows great potential^8,9^ in the early detection of AD spectrum disease.

Large-, structured neuroimaging databases (like Alzheimer’s Disease Neuroimaging Initiative (ADNI)) creates an opportunity to apply machine learning (ML) methods to analyse sMRI, fMRI data^10^ and differentiate MCI, AD patients and healthy volunteers with three inevitable steps in ML models^11^: (a) feature extraction (which converts the BOLD fMRI signals into relevant features (e.g. variables or characteristics)); (b) feature selection, where relevant features are selected for the classifier; (c) testing the different models. The most commonly used algorithms in classification of MRI data in AD and MCI are Support Vector Machines (SVM), Random Forests (RF) and artificial neural networks (ANN) including convolutional neural networks (CNN)^12–14^. ANN and CNN require larger amounts of data compared to SVM and RF algorithms. RF is not often used in fMRI studies compared to other methods. Previously it was utilized with other kind of metrics, e.g. for feature selection of self-assessed features in the diagnosis of MCI^15^. It was also used before on sMRI scans in a highly cited study^16^ with 90% accuracy, but its use in rs-fMRI based classification has not been explored^17^.

Feature selection is essential in fMRI studies because of the large number of features. Several studies used these techniques in the diagnosis of AD- and MCI patients. Bron et al., 2015 used the SVM weight vector to help with classification while reaching a 92% in terms of area under the receiver operating characteristic curve (ROC-AUC)^17^. Nguyen et al., used a hybrid multivariate pattern analysis for feature selection with an extreme learning machine classifier and managed to reach an almost perfect score of 98.57% classification accuracy on the ADNI database^18^. Lama et al., used a greedy score-based feature selection method where the classification accuracy of the SVM classifier increased from 78 to 80% compared to the absence of feature selection^19^. Moreover Zamani et al. utilized different evolutionary algorithms for feature selection achieving over 94% accuracy on early MCI patients from the ADNI database^21^. Altogether five feature selection algorithms were multiple time tested in MCI-AD classification.

While many studies applied feature selection and ML algorithms on the ADNI database^20,21^ these algorithms were not yet tested on smaller examples and/or at subject level. The aim of our study was to compare the efficiency of different classification algorithms and feature selection methods in the differentiation of MCI patients and healthy controls with data driven approach neglecting the neuropsychological or clinical scores. A local dataset was used to test feature selection algorithms and machine learning. To validate our results, we have applied the same algorithms on ADNI database and compared the results.

## Methods

### Local participants and neuropsychological examinations

78 participants were included in this study. Data were collected by two independent research centres: (1) the Semmelweis MCI Neuroimaging Cohort (SMNC) and the (2) AlzEpi Cohort Observational Library (ACOL). Data were harmonised under framework of the Euro-Fingers Consortium. Participants were recruited from the Department of Psychiatry and Psychotherapy, Semmelweis University (SMNC database), and from the National Institute of Mental Health, Neurology, and Neurosurgery (ACOL database). All subjects were native Hungarians.

Every participant underwent comprehensive neurological and neuropsychological evaluation carried out by neuropsychologists, neurologists, or trained neuroscientists. Furthermore, blood tests, CSF and MRI acquisition were completed. The neuropsychological test battery included the Hungarian version of the Rey Auditory Verbal Learning Test, the Hungarian version of the Addenbrooke’s Cognitive Examination, Trail-making Test A/B and Clinical Dementia Rating Scale. Beck Depression Inventory and Spielberger State and Trait Anxiety Inventory were used to assess the potential presence and level of depression and anxiety that could alter cognitive function. The healthy control group included participants with negative neurological status, no evidence of cognitive decline supported by the results of the neuropsychological tests, no clinically significant cortical atrophy, or brain lesions. The MCI group consisted of patients whose diagnosis was established according to the revised Petersen criteria^22^. Cognitive impairment was objectively determined based on the neuropsychological test results. Individuals who scored below the cut-off value in both the delayed recall subscore and the total score of the first five trials were categorized as belonging to the MCI group (Table 1). Structural MRI acquisition reinforced the presence of reduced total grey matter volume and showed decreased thickness of the entorhinal cortex. Based on these criteria, 46 individuals were classified as healthy controls, 20 subjects from the SMNC database and 26 participants from the ACOL database. The MCI group comprised 32 participants, 13 individuals from the SMNC database and 19 subjects from the ACOL database. Written consent was obtained from every participant. The Hungarian Medical Research Council authorized our research (reference number: 024505/2015 and IV/5831-3/2021/EKU).

**Table 1:** Applied age and education adjusted cut-off scores for the exclusion of dementia.

Education/age	50–54	55–59	60–64	65–69	70–74	75–79	80–84	85 +
MMSE cut off scores for the exclusion of dementia								
5–8 years	23	23	23	23	23	21	21	17
9–12 years	25	25	25	25	24	24	21	21
> 12 years	27	27	27	27	25	25	25	24

Age	Score							
RAVLT sum 5 cut-off scores								
50–59	39							
60–69	35							
70 +	29							

Age	Score							
RAVLT 7 cut-off scores								
50–59	6							
60–69	5							
70 +	4							

*MCI* mild cognitive impairment, *MMSE* mini mental state examination, *RAVLT* Rey auditory verbal learning task, *RAVLT SUM 5* the summarized number of learned words in the first five trials (max = 75), *RAVLT 7* number of recalled words after 30 delay (max = 15).

The study excluded individuals with dementia based on their age, education, and standardized Mini-Mental Examination Scores (MMSE) (Table 1). In addition, the study also applied further exclusion criteria that eliminated any participants with conditions that could potentially compromise their cognitive functions. These exclusion criteria included prior central nervous infection, clinically significant brain lesions such as cortical stroke, severe periventricular white matter disease, and white matter infarcts, head trauma with loss of consciousness, demyelinating conditions, hydrocephalus, untreated vitamin B12 deficiency, hypothyroidism, syphilis, HIV infection, major depression, schizophrenia, electroconvulsive therapy, renal insufficiency, liver disease, significant systemic medical illness, alcohol or substance dependency, and psychoactive drugs that could potentially affect cognitive functions.

### Participants from ADNI

155 participants (69 late MCI and 86-HC, age and sex matched) data were used from ADNI database. Within the ADNI database, a total of 185 participants were identified with late MCI. From this larger pool, our selection process focused on individuals who met specific imaging criteria (rs-fMRI imaging with the parameters that described in the MRI Examinations section) and 30 patients were excluded due to the differences in fMRI parameters. Patients were selected only if they corresponded to the same inclusion, exclusion criteria and the same neuropsychological scores and cutoffs what we applied to identify MCI patients. The preselection did not lead to exclusion, since our local MCI recognition protocol follows the system of ADNI. The ADNI database included data on 884 healthy subjects. In case of HC group, a random automated selection approach was applied matching with our local dataset in term of the sociodemographic parameters (age, sex and education) and the neuropsychological profile (average MMSE). Finally, a group of random 86 healthy participants matching our sample was generated for comparative analysis.

### Neuropsychological examination

Trained neuroscientists, neurologists, or neuropsychologists administered the neuropsychological tests. The MMSE test (maximum 30 points)^25^ was used to exclude patients with dementia, as it is the most used standard test in dementia research. While many studies have used a cut-off score of 26 to indicate clinically evident dementia, we used a widely accepted method that considers the educational background and age of the participants (Table 1)^26^.

The Hungarian version of the Addenbrooke Cognitive Examination (ACE)^27^ was used to assess global cognitive performance (maximum 100 points) and major cognitive subdomains, including orientation, attention, memory, verbal fluency, language, and visuospatial abilities. Although MCI patients typically have normal MMSE scores, studies suggest that ACE scores can already detect impaired cognitive performance^28^.

The Hungarian version of the Rey Auditory Verbal Learning Test (RAVLT)^29^ was used to objectively assess memory complaints according to the Petersen criteria. Previous studies have shown that RAVLT has excellent sensitivity in detecting MCI due to the early involvement of verbal-learning oriented memory functions^30^. Participants were asked to memorize a list of 15 words (list A) and recall them with five repetitions (RAVLT sum 5: immediate recalls described with the total number of correct words). They were then presented with another 15 words once (list B) followed by a recall. Later, they were asked to recall list A without repetition, and the same task was required 30 min later (RAVLT 7: delayed recall described with the total number of correct words).

The trail-making test (TMT) was used to measure executive functions and attention^31^. Test A required participants to connect numbers in ascending order, while test B (TMT-B) required them to connect numbers and letters in alphabetical order (1-A, 2-B, etc.). Results were described as the required time (in seconds).

### MRI examinations

All subjects underwent brain MRI, producing a high-resolution anatomical image, which is used for further processing analysis. At the National Institute of Mental Health, Neurology, and Neurosurgery, a Siemens Magnetom Verio 3 T scanner (Siemens Healthcare, Erlangen, Germany) was used with the standard 12 channels head receiver head coil. The protocol consisted of T1-weighted 3D MPRAGE (magnetization prepared rapid gradient echo) anatomical imaging (TR (time resolution) = 2.300 ms; TE (echo time) = 3.4 ms; TI = 100 ms; Flip Angle: 12°; Voxel Size: 1.0 × 1.0 × 1.0 mm). The second measurement was a resting-state functional MRI, an EPI-based MRI sequence (TR = 2000 ms; TE = 30 ms; Flip Angle = 79°; Voxel Size = 3 × 3 × 3 mm). The fMRI scan was 10 min long, while patients were laying on the table with closed eyes.

On the second site, image acquisitions were done at the MR Research Center, Semmelweis University on a 3 Tesla Philips Achieva whole-body MRI scanner (Philips Medical Systems, Best, The Netherlands) equipped with an 8-channel SENSE head coil. The high-resolution, whole-brain anatomical images were obtained using a T1 weighted three-dimensional spoiled gradient echo (T1W 3D Turbo Field Echo) sequence. About 180 contiguous slices were acquired from each subject with the following imaging parameters: TR = 9.7 ms; TE = 4.6 ms; flip angle = 8°; FOV (field-of-view): 240 mm × 240 mm; voxel size: 1.0 × 1.0 × 1.0 mm. The “resting-state” part of the fMRI acquisition took 8.5 min. During that time, subjects were instructed to fixate on a cross in the centre of the screen. Subjects were briefed on whether they fell asleep during the recording process, and no subject reported doing so. Head motion was minimised using foam padding. Functional images were acquired using a T2* weighted echo-planar imaging (EPI) sequence with the following parameters: TR = 2 s; TE = 30 ms; Flip Angle = 70°, FOV: 240 mm × 240 mm; Voxel Size: 3.0 × 3.0 × 4.0 mm; Number of Slices = 36.

Both protocols consisted of a T2-, diffusion-, and a FLAIR-weighted sequence to identify the possible pathological lesions.

The ADNI dataset comprised of multiple different MRI scans with very similar protocols. The sMRI scans were the same for all subjects: 256 × 256 × 170 voxels and 1 × 1 × 1 mm^3^. Resting-state fMRI scans were performed on a 3 T Philips scanner with the following parameters: Field Strength = 3 T; Flip Angle = 80.0°; Matrix = 64 × 64 pixels; Pixel Spacing = 3.3 mm; Slice Thickness = 3.3 mm; TE = 30.0 ms; TR = 3000.0 ms. For the 3 T Siemens scanner the scan parameters are: Field Strength = 3 T; Flip Angle = 90°; Matrix = 448 × 448 pixels; Pixel Spacing = 3.4 mm; Slice Thickness = 3.4 mm; TE = 30.0 ms; TR = 3000.0 ms. For the 3 T GE scanners the image characteristics are: Field Strength = 3.0 T; Flip Angle = 90°; Matrix = 64 × 64 pixels; Pixel Spacing = 3.3 mm; Slice Thickness = 3.3 mm; TE = 30.0 ms; TR = 2925.0 ms.

### fMRI image preprocessing

CONN toolbox^32^ was used for resting-state fMRI data analysis. We applied the standard fMRI preprocessing pipeline, which includes functional realignment and unwarp, slice-time correction (interleaved at Siemens’ scanner data ascending at Philipps’ scanner data), outlier detection (ART-based identification of outlier scans for scrubbing), direct functional and structural segmentation, normalisation (simultaneous Gray/White/CSF segmentation and MNI normalisation), and spatial smoothing. After the preprocessing, we ran an additional quality check to quantify the segmentation accuracy. Band-pass filter was applied between 0.008 and 0.09 Hz to eliminate the physiological-based artefacts and the unrelated part of the measured signal. Finally, linear regression was used to filter out/eliminate white matter, CSF signal, and the effect of realignment and scrubbing.

### rs-fMRI metrics

Three voxel-based metrics were used from the CONN Toolbox: Intrinsic Connectivity (ICC), Local Correlation (LCOR) and Fractional Amplitude of Low Frequency Fluctuations (fALLF). All three measures and their similar variants were previously used in different neuropsychiatric conditions^33–35^. ICC was used to investigate the interconnectedness of different brain regions. It shows how strong the connectivity of a voxel is to all other voxels. How many other voxels are connected to a voxel at a certain threshold value^27^. Local connectivity between brain regions was calculated with LCOR. It shows the local coherence of each voxel. It depicts a voxels’ connectivity with other voxels in adjacent areas where the degree of adjacency will be given by a Gaussian weight function^36^. In our case we used the default parameter for Gaussian function which was 25 mm. To assess the magnitude of the signals fALLF measure was used, which reflects the neural activity of each brain voxel^36^^.^

### Feature selection and classification

To define the ROI, the default atlas in CONN Toolbox was used to achieve the highest possibility for further comparisons of the study results. The Toolbox combines the FSL Harvard–Oxford atlas at cortical and subcortical areas and the AAL atlas at cerebellar regions. It means a total of 132 regions of interest (ROIs). The mean values for each ROI were then extracted from the maps to create a feature vector with 132 dimensions for each map. Four evolutionary- and one sequential feature selection algorithm was used to find the most efficient set of features with different selection criteria. The algorithms and the selection criteria were as follows:

Genetic Algorithm (GA): GA is based on natural genetics and biological evolution and consists of 5 main steps: population initialization, fitness function evaluation, parent selection, gene crossing, mutation^37^. The selection criteria were the accuracy of the ML models. The algorithm was implemented from the sklearn Python library.

Non-dominated Sorting Genetic Algorithm II (NSGA-II): NSGA is a multi-objective optimization algorithm which captures multiple optimal solutions simultaneously. The sorting of new members is based on non-dominant sorting and crowding (crowding distance)^38^. Since this is a multi-objective optimization algorithm, we used two functions for the selection criteria. The first one was the number of features. The second one was Eq. (1).

1 $$f\left(x\right)=\alpha *\left(1-P\right)+\left(1-\alpha \right)*\frac{{N}_{selected}}{{N}_{total}}$$

where *α* is a parameter that decides the trade-off between the classifier performance *P* and the number of features selected. The algorithm was implemented from the Pymoo Python library.

Particle Swarm Optimization: A stochastic optimization method that utilises the swarming behaviour of animals. Each member finds optimal regions of the search space by coordinating with other particles in the population^39^. The selection criteria were the second function in the NSGA-II section. The algorithm was implemented from the NiaPy Python library.

Simulated Annealing (SA): SA is a stochastic search algorithm. A new feature subset is selected randomly with each iteration^40^. Here we used the accuracy of the model as our selection criteria. The algorithm was implemented by home-based codes.

Sequential Floating Forward Selection: In the SFFS algorithm we iteratively add or remove features from a subset of the original feature set based on the model performance with the increment of one feature at a time^41^. The selection criteria were the accuracy of the models. The algorithm was implemented from the mlextend Python library.

For classification SVM^42^ and the RF^43^ algorithm was implemented from the sklearn Python library. Both classification and feature selection were performed via a tenfold cross validation which was implemented from the sklearn Python library. The best subset of features was selected by maximizing the selection criteria for each algorithm that was calculated from the mean of the 10 folds.

For each final subset produced by each algorithm, we calculated various performance metrics, including accuracy, sensitivity, specificity, ROC-AUC score and confusion matrix (Table 2). The confusion matrix depicts the true positive (TP), true negative (TN), false positive (FP), and false negative (FN) values. All four metrics and the confusion matrix were calculated from the tenfold cross-validation. For the accuracy, sensitivity, and specificity we used the matrix to calculate the metrics. The ROC-AUC score was calculated by taking each value from the cross validation and calculating the mean. These metrics allowed us to evaluate the effectiveness of the feature selection methods in differentiating between the two groups.

**Table 2:** Structure of the confusion matrix.

		Predicted	
		HC	MCI
Reference data	HC	TN	FP
	MCI	FN	TP

*TP* true positive: the number of participants that were correctly classified in the MCI group, *FP* false positive: the number of participants that were incorrectly classified in the MCI group, false negative *FN* false positive: the number of participants that were incorrectly classified in the HC group, *TN* true negative, the number of participants that were correctly classified in the HC group, *MCI* mild cognitive impartment, *HC* healthy controls.

Accuracy, sensitivity, and specificity were calculated, using the conventional formulas (2–4).

2 $$accuracy=\frac{TP+TN}{TP+TN+FP+FN}$$

3 $$sensitvity=\frac{TN}{TN+FN}$$

4 $$specificity=\frac{TP}{TP+FN}$$

**Figure 1:**
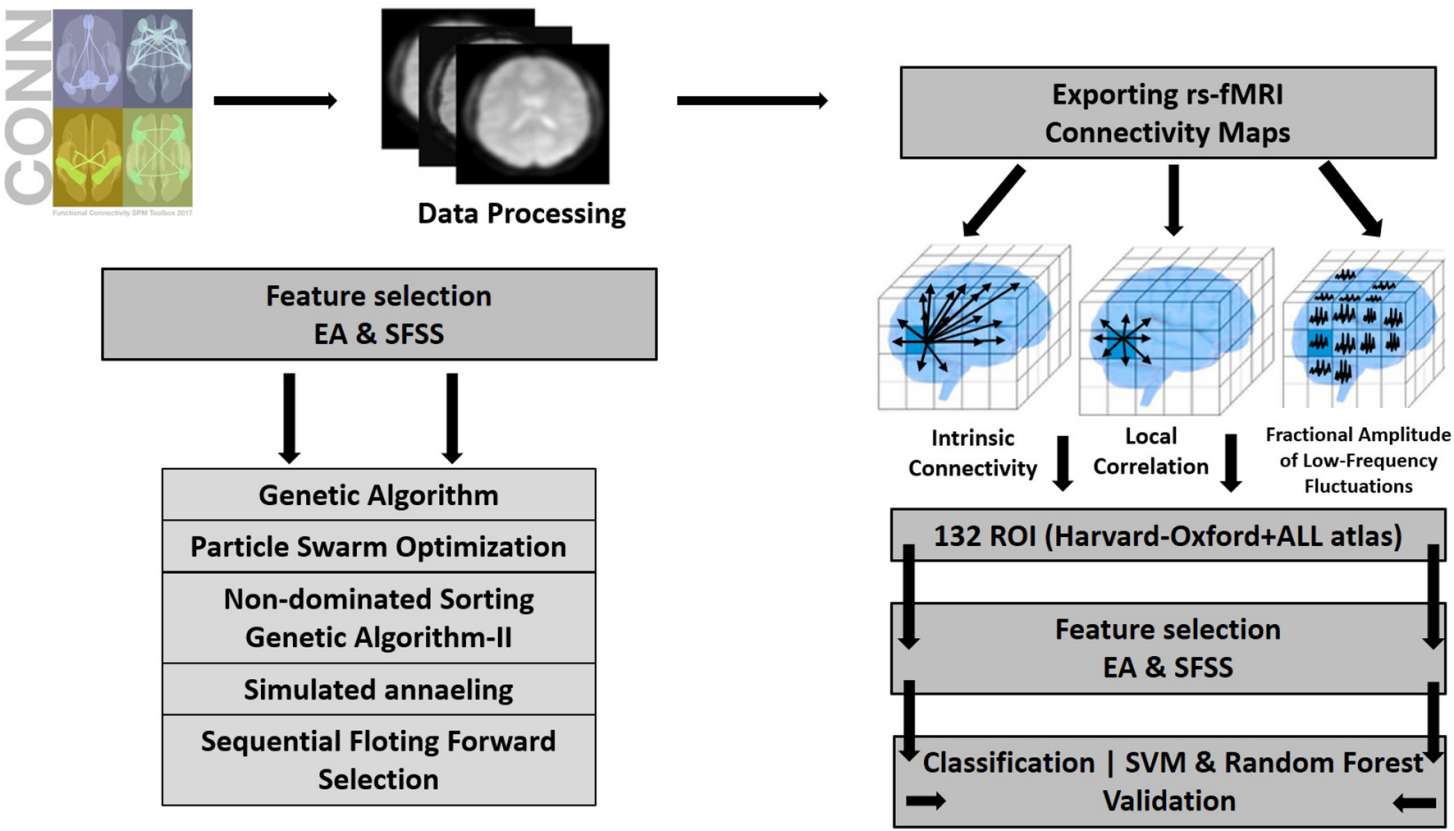
Pipeline of the procedure: full analysis procedure of the feature extraction, feature selection and classification based on rs-fMRI data. CONN Toolbox was used to analyse rs-fMRI data from 78 participants (n = 32 MCI; n = 46 HC). Preprocessing, feature extraction of three rs-fMRI metrics (ICC, LCOR, fALFF) was done. Then we calculated the average of each metric for different brain regions (132 regions based on ALL and Harvard–Oxford atlas). The extracted parameters (3 × 132 brain regions) were subsequently given to one of the feature selection methods to determine the best subset of ROIs for classification. Five feature selection methods were analysed consisting of 4 optimization and one conventional feature selection algorithm. Two classification algorithms were utilized (SVM, RF) with the algorithms. *EA* evolutionary algorithm, *SFFS* sequential floating forward selection, *SVM* support vector machine, *ICC* intrinsic connectivity, *LCOR* local correlation, *fALFF* fractional amplitude of low frequency fluctuations.

The whole study pipeline is summarized in Fig. 1.

## Results

### Demographics and Cognitive Performance

The study groups differed in age and sex ratio, but there was no significant difference in education length between the HC and MCI groups (p = 0.142). The HC group had a higher proportion of female participants (chi-square test:χ^2^ = 5.128; p = 0.024). The MCI group consisted of a significantly older study population (F = 6.18; p = 0.015).

Significant differences were found in the total scores of all neuropsychological tests (Table 3), with many of them surviving the application of Benjamini–Hochberg correction. The MCI group performed worse than controls on several measures, including MMSE score (F = 9.098; p < 0.001), total ACE score (F = 11.065; p < 0.001), RAVLT sum-5 score (F = 13.53; p < 0.001), and RAVLT 7 score (F = 11.9; p < 0.001). Additionally, MCI patients had longer completion times for TMT-A (F = 4.69; p = 0.048) and TMT-B (F = 5.51; p = 0.021), indicating weaker cognitive performance. Controls outperformed MCI patients in VS skills (F = 8.32; p < 0.001), but no significant differences were found in other cognitive subdomains when corrected for multiple comparisons (p > 0.05). Age and sex did not significantly affect the neuropsychological results (p > 0.05).

**Table 3:** Demographic and neuropsychological characteristics of study groups.

	HC (n = 46)	MCI (n = 32)	p-value	Effect size
Demographics				
Age (years)	67.63 ± 7.15	70.68 ± 9.94	0.015	0.352
Sex (% of females)	69.6	53.1	0.024	–
Education (years)	15 ± 2.53	14.43 ± 3.13	0.142	0.2
Neuropsychology				
MMSE	28.52 ± 1.13	26.87 ± 1.62	p < 0.001	1.181
ACE total	93.24 ± 3.29	82.31 ± 7.26	p < 0.001	1.939
ACE orientation	9.88 ± 0.31	82.31 ± 7.26	0.029	0.742
ACE attention	7.91 ± 0.28	7.65 ± 0.86	0.134	0.407
ACE memory	30.97 ± 2.11	24.68 ± 5.47	0.03	1.517
ACE verbal fluency	11.95 ± 2.24	9.62 ± 2.87	0.021	0.905
ACE language	27.71 ± 0.54	27.125 ± 1.58	0.378	0.495
RAVLT sum-5	48.43 ± 8.69	31.15 ± 9.4	p < 0.001	1.909
RAVLT 7	9.89 ± 2.75	4.03 ± 2.83	p < 0.001	2.1
TMT-A	39.62 ± 10.58	90.41 ± 66.98	0.008	1.059
TMT_B	83.13 ± 32.67	209.33 ± 147.31	0.003	1.183

Data is in mean ± standard deviation form. Sex: % of female participants in the groups, P is in nominal form, p < 0.05 was used as the threshold for determining statistical significance after Benjamini–Hochberg correction. Effect size is in Cohen’s d (0.2–0.5 = small, 0.5–0.8 = medium, > 0.8 = large). Age was analyzed with independent sample t-test, Sex was analyzed with chi-square test, education was analyzed with Mann–Whitney U-test. The Neuropsychology results were analyzed with ANCOVA with age and sex as covariates.

*MCI* mild cognitive impairment, *HC* healthy control, *MMSE* mini mental state examination, *RAVLT* Rey auditory verbal learning task, *ACE* Addenbrooke cognitive examination, *TMT* trail making test.

### Selected features

**Figure 2:**
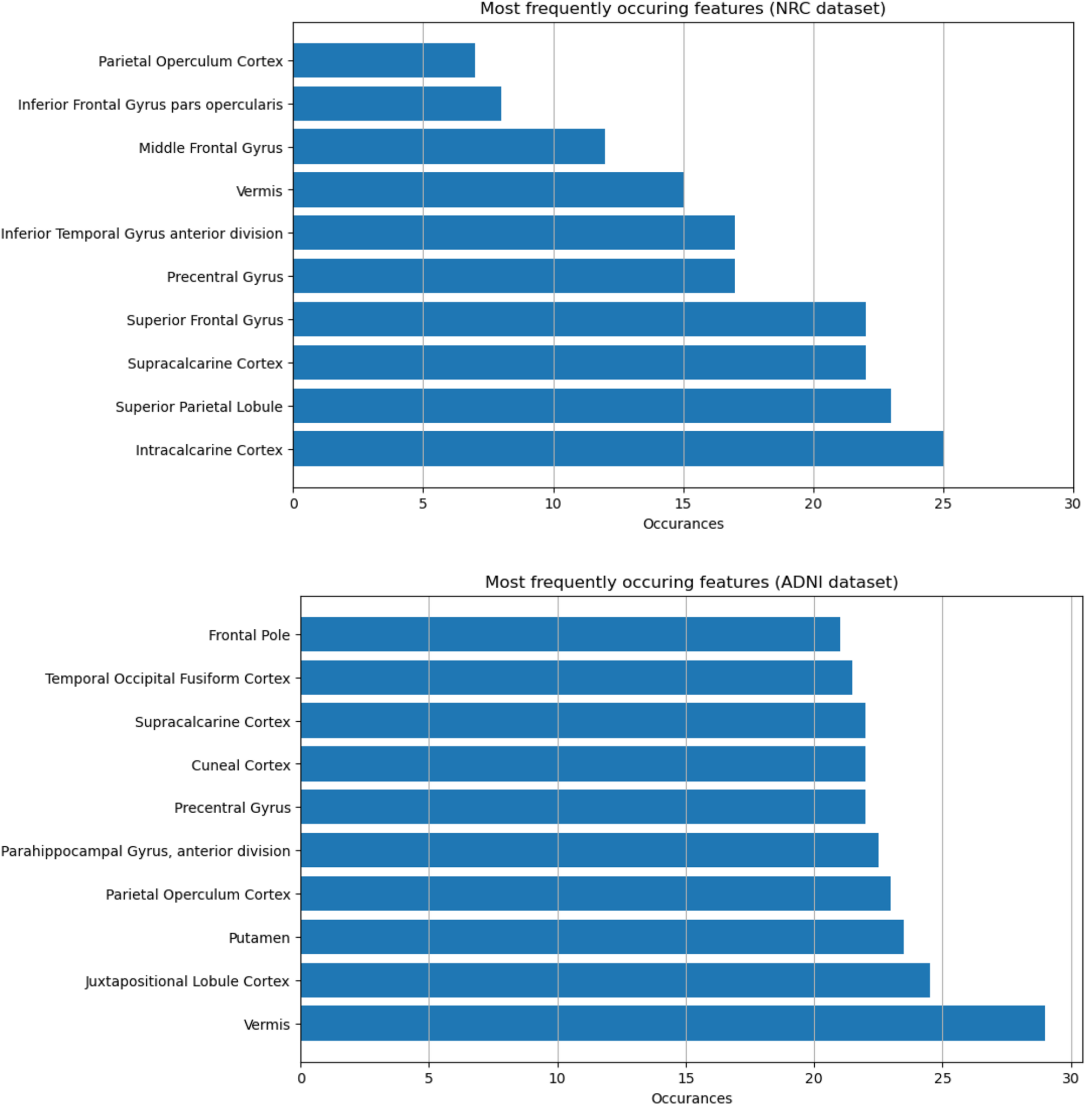
Most frequent anatomical regions selected by the algorithms: shows the regions that were most frequently selected by the feature selection algorithms. The five most frequently appearing ones are: Intracalcarine Cortex, Superior Parietal Lobule, Superior Frontal Gyrus, Supracalcarine Cortex, Inferior Temporal Gyrus (anterior division) and the Precentral Gyrus. Occurrences: The number of times one feature appeared in selected features by the five algorithms.

To identify the relevant features in the classification process we extracted the most frequently appearing anatomical regions over all five feature-selection and two classification algorithms (Fig. 2). The most indicative features from our dataset are: Intracalcarine Cortex, Superior Parietal Lobule, Superior Frontal Gyrus, Supracalcarine Cortex, Inferior Temporal Gyrus (anterior division) and the Precentral Gyrus. From the ADNI dataset the most frequently occurring regions are Vermis, Juxtapositional Lobule, Parietal Operculum Cortex, Putamen, Parahippocampal Gyrus and the Precentral Gyrus.

### SVM classifier

**Figure 3:**
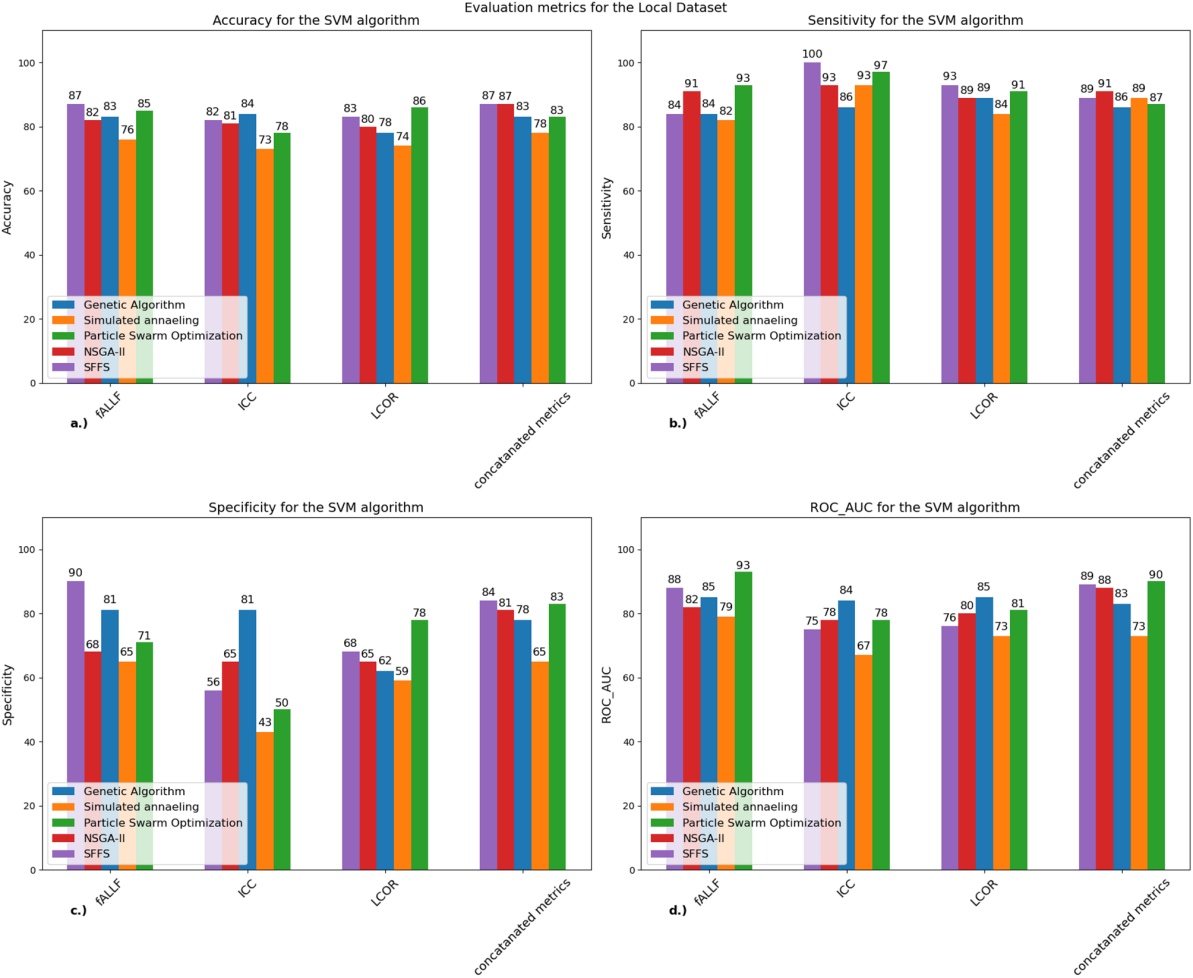
Performance metrics of the SVM classifier for all five algorithms for our local dataset: A bar chart that shows (**a**) the mean accuracy, (**b**) sensitivity, (**c**) specificity and (**d**) ROC-AUC scores for each algorithm calculated from the tenfold cross-validation for the SVM classifier. The best results were acquired when we combined the three metrics where two algorithms (SFFS, NSGA-II) managed to achieve 87% accuracy. *SVM* support vector machine, *ICC* intrinsic connectivity, *LCOR* local correlation, *fALFF* fractional amplitude of low frequency fluctuations, *SFFS* sequential floating forward selection, *NSGA-II* non-dominated sorting genetic algorithm concatenated metrics: the three metrics combined resulting in 396 (3 × 132) regions.

We investigated the performance of the five evolutionary algorithms on all three rs-fMRI metrics, then combined three of them. Figure 3 and Table 4 depict the SVM classifiers performance metrics and their values with the five algorithms in all metrics at our local dataset.

**Table 4:** Confusion matrix for the SVM algorithm with concatenated metrics (local dataset): confusion matrices that show the performance of the GA, SFFS, NSGA-II, PSO algorithms when used with the SVM classifier.

GA			SFFS			NSGA-II			PSO		
Confusion matrix	Sensitivity	Specificity	Confusion matrix	Sensitivity	Specificity	Confusion matrix	Sensitivity	Specificity	Confusion matrix	Sensitivity	Specificity
$$\left[\begin{array}{cc}43& 3\\ 8& 24\end{array}\right]$$	93%	75%	$$\left[\begin{array}{cc}45& 1\\ 9& 23\end{array}\right]$$	97%	72%	$$\left[\begin{array}{cc}42& 4\\ 6& 26\end{array}\right]$$	91%	81%	$$\left[\begin{array}{cc}42& 4\\ 8& 24\end{array}\right]$$	95%	75%

The table also shows the sensitivity and specificity values for each depicted algorithm.

*GA* genetic algorithm, *SFFS* sequential floating forward selection, *NSGA-II* non-dominated sorting genetic algorithm II, *PSO* particle swarm optimization, *SVM* support vector machine.

**Figure 4:**
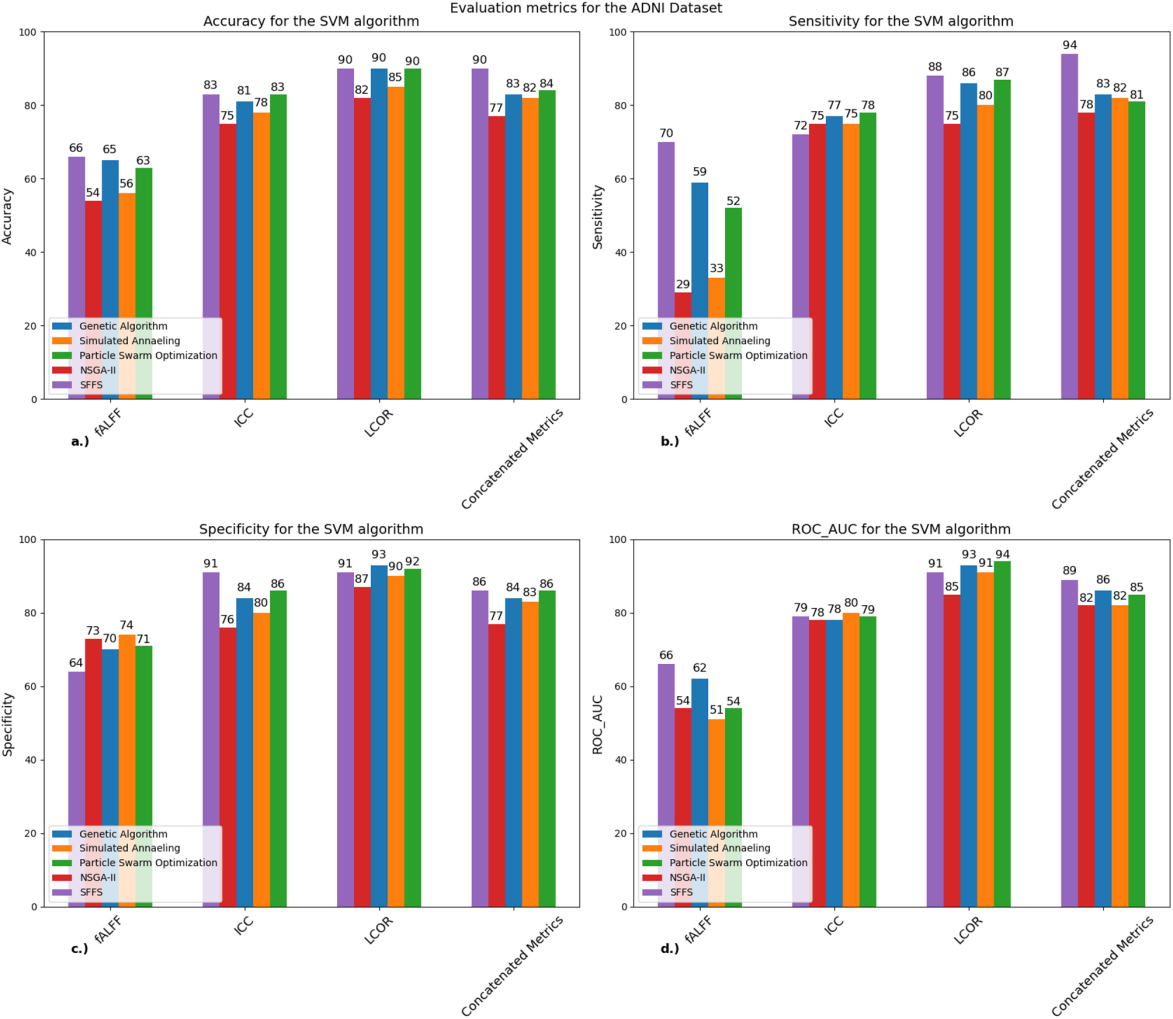
Performance metrics of the SVM classifier for all five algorithms for the ADNI dataset: a bar chart that shows (**a**) the mean accuracy, (**b**) sensitivity, (**c**) specificity and (**d**) ROC-AUC scores for each algorithm calculated from the tenfold cross-validation for the SVM classifier. The best results were acquired with the LCOR metric (90%). *SVM* support vector machine, *ICC* intrinsic connectivity, *LCOR* local correlation, *fALFF* fractional amplitude of low frequency fluctuations, *SFFS* sequential floating forward selection, *NSGA-II* non-dominated sorting genetic algorithm concatenated metrics: the three metrics combined resulting in 396 (3 × 132) regions.

We achieved the best and most consistent accuracy (87%) when combining all three metrics. However, most of the performances were above 80%. We can also see that the SA algorithm was the worst performing out of all cases. To assess the efficiency of the models we also calculated the mean ROC AUC values (Fig. 4) where the highest scores were achieved when combining all three metrics together.

Table 4 shows the confusion matrices of the four best performing algorithms with concatenated metrics.

The confusion matrices show that there are mostly false negative values even with the best performing algorithms. The NSGA-II algorithm managed to get the best score with a good accuracy, and it also reached the highest specificity value from all algorithms.

Figure 4 shows the performance metrics for the SVM classifier at ADNI dataset.

In case of the ADNI dataset there are higher accuracy scores compared to our local dataset. The LCOR metric outperformed other metrics where three algorithms achieved 90% accuracy, although we can see above 80% accuracy in other metrics as well. Also, the SFFS algorithm managed to reach 90% accuracy in the concatenated metrics section. However, this section did not show the same improvement as in our local dataset. The specificity and ROC-AUC values also indicate that the LCOR metric was more effective in discriminating the LMCI group from the controls (Table 5).

**Table 5:** Confusion matrix for the SVM algorithm for different rs-fMRI metrics (ADNI dataset): confusion matrices that show the performance of the GA, SFFS, PSO algorithms when used with the SVM classifier.

GA-LCOR			SFFS-LCOR			PSO-LCOR			SFFS-concatenated metrics		
Confusion matrix	Sensitivity	Specificity	Confusion matrix	Sensitivity	Specificity	Confusion matrix	Sensitivity	Specificity	Confusion matrix	Sensitivity	Specificity
$$\left[\begin{array}{cc}80& 6\\ 10& 59\end{array}\right]$$	93%	85%	$$\left[\begin{array}{cc}78& 8\\ 8& 61\end{array}\right]$$	90%	88%	$$\left[\begin{array}{cc}79& 7\\ 9& 60\end{array}\right]$$	91%	86%	$$\left[\begin{array}{cc}74& 12\\ 4& 65\end{array}\right]$$	89%	94%

The table also shows the sensitivity and specificity values for each depicted algorithm.

*GA* genetic algorithm, *SFFS* sequential floating forward selection, *NSGA-II* non-dominated sorting genetic algorithm II, *PSO* particle swarm optimization, *SVM* support vector machine.

Table 5 shows the confusion matrices of the three best performing algorithms for the ADNI dataset for the different rs-fMRI metrics.

The confusion matrices in this case also show that there were more false negative values. One exception is the SFFS algorithm with concatenated metrics where there were more false positive values compared to false negative. The specificity was also the highest in this case.

### Random forest classifier

**Figure 5:**
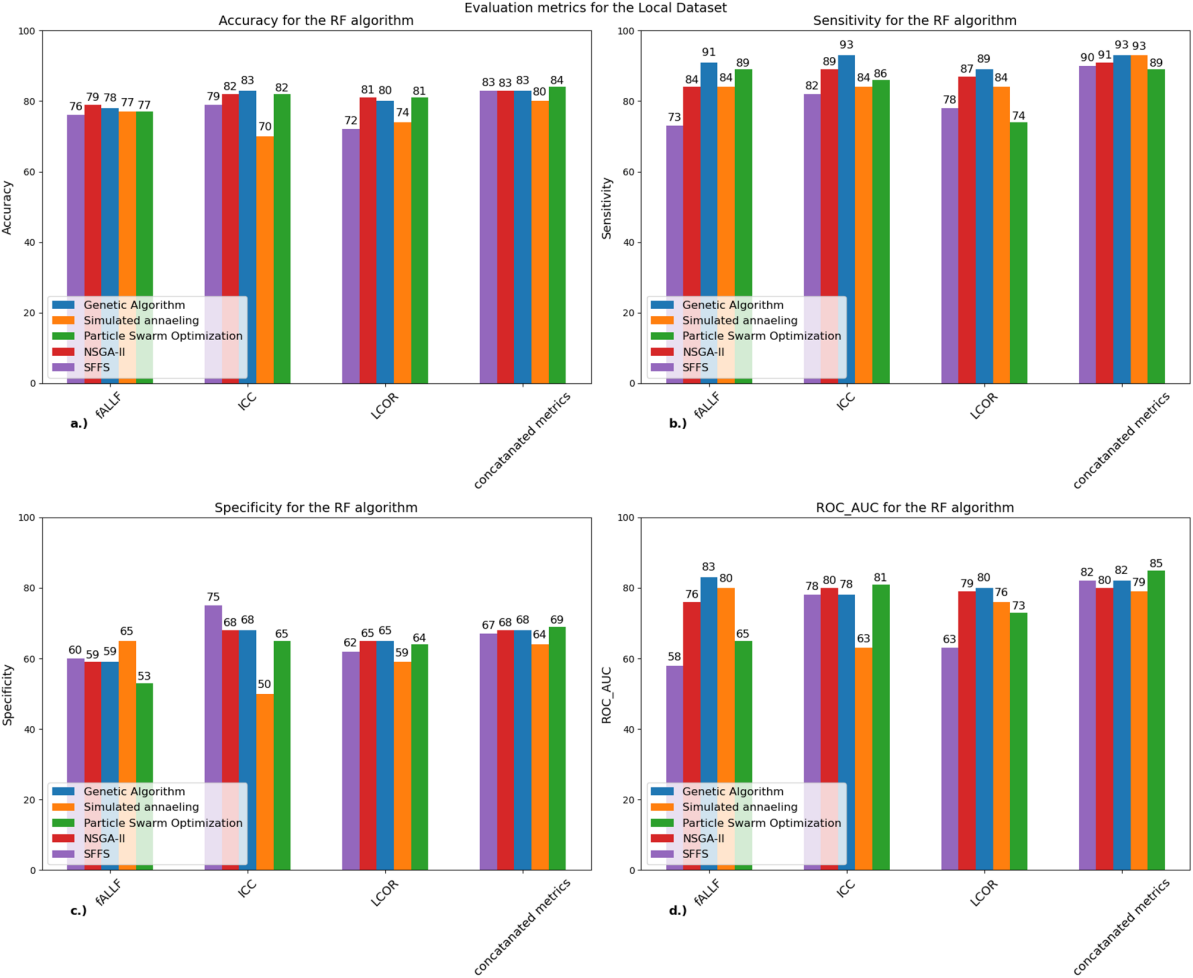
Performance metrics of the RF classifier for all five algorithms for our local dataset: A bar chart that shows (**a**) the mean accuracy, (**b**) sensitivity, (**c**) specificity and (**d**) ROC-AUC scores for each algorithm calculated from the tenfold cross-validation for the RF classifier. The best and results were acquired when we combined the three metrics where multiple algorithms (GA, NSGA-II, GA, PSO) managed to achieve 83% accuracy. *RF* random forest, *ICC* intrinsic connectivity, *LCOR* local correlation, *fALFF* fractional amplitude of low frequency fluctuations, *SFFS* sequential floating forward selection, *NSGA-II* non-dominated sorting genetic algorithm concatenated metrics: The three metrics combined resulting in 396 (3 × 132) regions.

Figure 5 and Table 6 depict the performance metrics of the RF algorithm for our local dataset.

**Table 6:** Confusion matrix for the RF algorithm with concatenated metrics (local dataset)*:* confusion matrices that show the performance of the GA, SFFS, NSGA-II, PSO algorithms when used with the RF classifier.

GA			SFFS			NSGA-II			PSO		
Confusion matrix	Sensitivity	Specificity	Confusion matrix	Sensitivity	Specificity	Confusion matrix	Sensitivity	Specificity	Confusion matrix	Sensitivity	Specificity
$$\left[\begin{array}{cc}43& 3\\ 8& 24\end{array}\right]$$	93%	75%	$$\left[\begin{array}{cc}42& 4\\ 9& 23\end{array}\right]$$	91%	72%	$$\left[\begin{array}{cc}44& 2\\ 11& 21\end{array}\right]$$	95%	65%	$$\left[\begin{array}{cc}44& 2\\ 9& 23\end{array}\right]$$	95%	72%

The table also shows the sensitivity and specificity values for each depicted algorithm.

*GA* genetic algorithm, *SFFS* sequential floating forward selection, *NSGA-II* non-dominated sorting genetic algorithm II, *PSO* particle swarm optimization, *RF* random forest.

Highest accuracy was achieved when we used the three fMRI metrics together. However, we only achieved 84% accuracy, the results were more homogeneous in the other metrics compared to the SVM algorithm. The specificity scores were low meaning that the RF algorithm also couldn’t classify the MCI group well. The ROC AUC scores were also the highest and most consistent in the combined metrics column.

Table 6 shows the confusion matrices of the four best performing algorithms with concatenated metrics.

Table 6 depicts that the RF algorithm also classified HC correctly, however it was worse at detecting MCI than the SVM algorithm. PSO algorithm had the highest accuracy with the best sensitivity and specificity values, but it was still worse at classifying the MCI group correctly.

**Figure 6:**
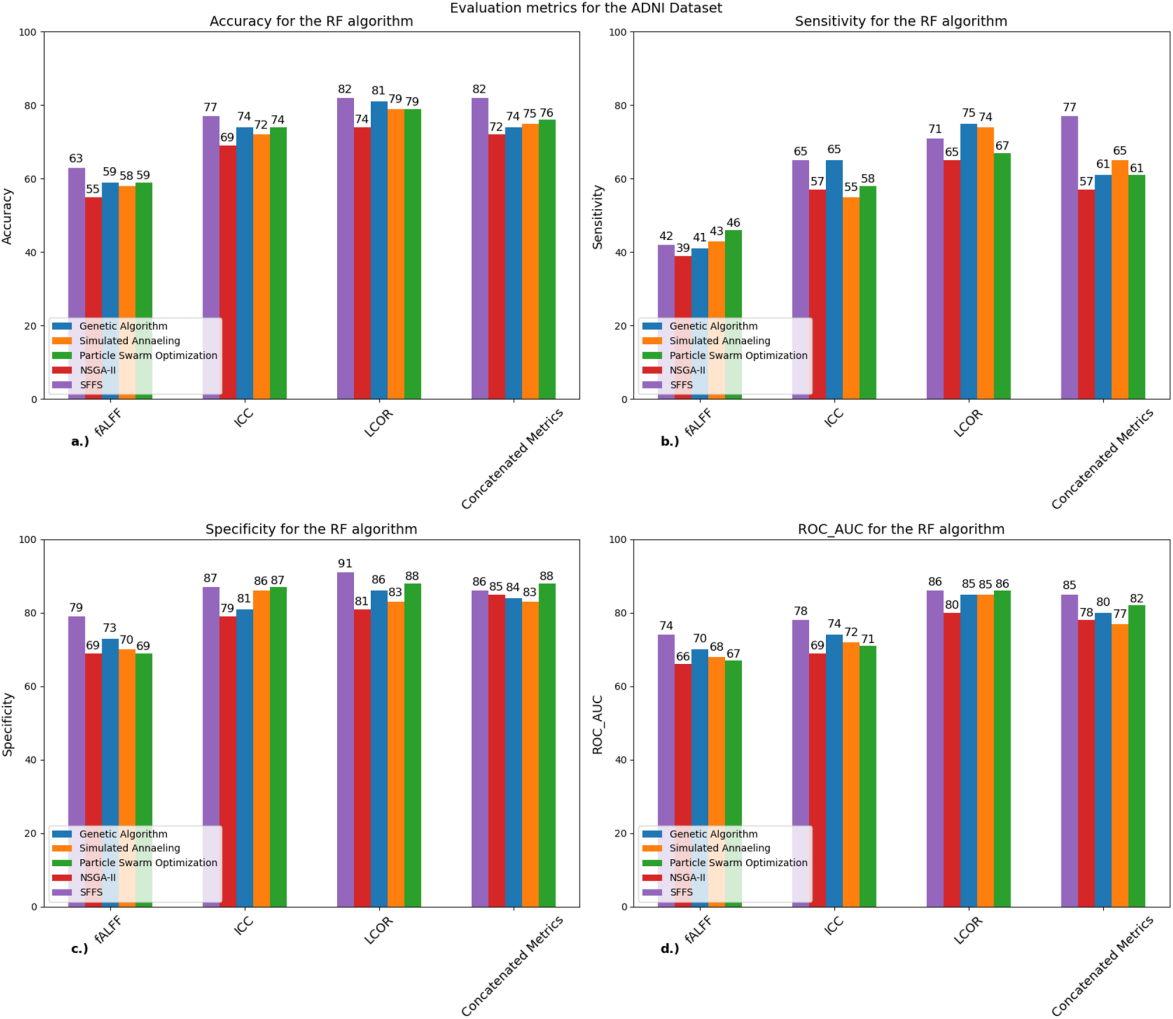
Performance metrics of the RF classifier for all five algorithms for the ADNI dataset: A bar chart that shows (**a**) the mean accuracy, (**b**) sensitivity, (**c**) specificity and (**d**) ROC-AUC scores for each algorithm calculated from the tenfold cross-validation for the SVM classifier. The best and most consistent results were acquired with the LCOR metric (82%). *SVM* support vector machine, *ICC* intrinsic connectivity, *LCOR* local correlation, *fALFF* fractional amplitude of low frequency fluctuations, *SFFS* sequential floating forward selection, *NSGA-II* non-dominated sorting genetic algorithm concatenated metrics: the three metrics combined resulting in 396 (3 × 132) regions.

Figure 6 shows the performance metrics for the RF classifier at ADNI dataset.

In this instance there is a similar pattern to our local example. The accuracy values were lower compared to the SVM algorithm but also more consistent across the feature selection algorithms. The specificity values were higher than our example but still lower compared to the SVM algorithm when tested with the ADNI dataset. A notable difference compared to our local case is that the LCOR metric also reached the highest score in this case just like with the SVM algorithm. The ROC-AUC values were also the highest in the LCOR metric.

Table 7 shows the confusion matrices of the four best performing algorithms for the ADNI dataset with different rs-fMRI metrics.

**Table 7:** Confusion matrix for the RF algorithm for different rs-fMRImetrics (ADNI dataset): confusion matrices that show the performance of the GA, SFFS, algorithms when used with the RF classifier.

GA-LCOR			SFFS-LCOR			SFFS-concatenated metrics		
Confusion matrix	Sensitivity	Specificity	Confusion matrix	Sensitivity	Specificity	Confusion matrix	Sensitivity	Specificity
$$\left[\begin{array}{cc}74& 12\\ 17& 52\end{array}\right]$$	86%	75%	$$\left[\begin{array}{cc}78& 1\\ 20& 49\end{array}\right]$$	90%	71%	$$\left[\begin{array}{cc}74& 12\\ 16& 53\end{array}\right]$$	95%	65%

The table also shows the sensitivity and specificity values for each depicted algorithm.

*GA* genetic algorithm, *SFFS* sequential floating forward selection, *NSGA-II* non-dominated sorting genetic algorithm II, *PSO* particle swarm optimization, *RF* random forest.

The matrices also show similarity with the two datasets. The RF algorithm also has lower specificity values, and the confusion matrices show that there were mostly false negative values. Just like the SVM algorithm with the ADNI dataset the SFFS algorithm with concatenated metrics had the lowest specificity value (Table 7).

## Discussion

The aim of this study was to differentiate MCI patients and healthy controls based on three rs-fMRI metrics on two independent datasets: a local Hungarian dataset from two research centres and a larger international dataset (ADNI database). To achieve this goal, we compared five feature selection and two machine learning algorithms (SVM, RF). We managed to achieve above 80% accuracy on both our local- and the ADNI dataset with both classification and most feature selection algorithms. On our local dataset we reached 87% accuracy and achieved 90% on the ADNI database. Both high scores were achieved with the SVM classifier.

In the case of our local dataset the best results regarding both the accuracy and the ROC-AUC scores were acquired when we combined the three metrics indicating that each metric contained important information. In this case the SA algorithm could not perform as well as the others, probably due to that this algorithm chooses its’ features randomly and does not factor in a selection criterion. The other algorithms generally perform better and achieve overall good scores (above 80% accuracy). The SFFS NSGA-II and PSO algorithms consistently achieved an above 80% classification accuracy in all metrics considering our case and the dataset from ADNI database. The SFFS algorithm is capable of evaluating a large number of features with a selection criterion always choosing the best model with the increment of one feature. This is more computationally expensive, however; achieves good performances. The NSGA-II algorithm utilises two functions to minimise and gives multiple good solutions for a single problem. This might be useful in many cases since its multi-objective nature could achieve good score consistently. In the PSO algorithm one particle’s next position is influenced not only by their own but also the overall global best position of all particles and some randomness. For this reason, it is also able to traverse large search spaces and find the best solution for the given problem. The same pattern in both the RF and SVM cases arises.

The results show positive and similar patterns across the two datasets, but with some differences. For our local dataset, the best outcome was achieved by combining all features. For the ADNI dataset, the best outcome was obtained by using the LCOR metric. The difference in the optimal metric could be due to the sample size, as our local dataset had only 78 participants and may have required more information from all three metrics, while the ADNI dataset had enough samples to discriminate the groups based on the LCOR metric alone. The reason why LCOR reached such high scores is that it may be more robust to noise and artefacts than other metrics, such as ICC or fALFF, because it averages over a local neighbourhood of voxels, which may reduce the influence of outliers or false correlations. However, the SFFS algorithm also achieved 90% on the concatenated metrics section with the ADNI dataset, indicating that the other metrics also carry important information. The fALFF metric did not reach 80% accuracy on the ADNI dataset with any classifier. A possible explanation is that while introducing more samples can help the algorithms generalize better but it can also induce some variance and heterogeneity which can decrease the performance in certain metrics. The ADNI dataset also had more balanced sensitivity and specificity values, and many 90% specificity values, indicating a higher accuracy in predicting the MCI group than the local dataset. This could be attributed to the greater number of participants in the ADNI dataset. We also achieved multiple 90% accuracy scores on the ADNI while on our example the best score was 87%.

Classifying data into groups of MCI and HC is proven to be a more difficult task than classifying AD and HC. Yet, few research groups have succeeded in achieving a classification accuracy above 90%. Most of these studies use convolutional neural networks and other deep neural networks. Nevertheless, these algorithms require extensive amounts of input data. One study used 755 HC and 755 MCI reaching 92% accuracy^44^. Another study used 209 HC and 384 MCI patients and got 98% accuracy^45^. Using our smaller sample size dataset, we managed to achieve 87% accuracy with our SVM classifier.

One other way to increase the model’s performance is to use different modalities. For example, a combination of PET, MRI and neuropsychology can be used to improve performance compared to PET or MRI only^46^. A recent study showed that DTI and MRI can improve the accuracy of each other by 20%^47^. In our current study we used rs-fMRI only which is a single modality and still managed to perform relatively well.

Interpretability is also important. When using deep neural networks or convolutional neural networks it can be hard evaluating how models arrive at their conclusions. They are so-called “black box” models which are difficult to verify. Our approach uses machine learning models for the classification and basic optimization algorithms for the feature selection. Thus, the model’s input features are understandable so it is easier to verify why that brain region might have been selected.

In this current study we looked at the six most frequently occurring regions chosen by the algorithms in both cases (Fig. 2). We obtained the best results when we combined all three metrics with our local dataset, and the best performing models also contain at least one of these ROI-s. In our local dataset the regions include the Intracalcarine Cortex, Superior Parietal Lobule, Superior Frontal Gyrus, Supracalcarine Cortex, Inferior Temporal Gyrus and the Precentral Gyrus. The most often occurring ROIs correspond to the frontotemporal and parietal regions being in line with recent studies suggesting that these regions are the most often affected by AD pathology^48–50^. Furthermore, a recent fMRI study on ADNI patients highlighted these regions as the most indicate for the identification of MCI^21^. In the ADNI dataset the most frequently occurring regions were Vermis, Juxtapositional Lobule (previously known as supplementary motor area involving the superior frontal gyrus), Parietal Operculum Cortex, Putamen, Parahippocampal Gyrus and the Precentral Gyrus. Most of the regions selected are also from the frontotemporal and parietal regions being in line with the previous observation in MCI^49,50^. There are also some exact overlaps with our regions such as the Supracalcarine Cortex, Superior Frontal Gyrus and the Precentral Gyrus. A possible explanation for the indicative role of these regions is the early impairment of dorsal attention network^51^ and the disconnection of this network and default mode network^52,53^ in MCI pathology.

One interesting region amongst the most frequent features in both cases is the Precentral Gyrus. A possible explanation for its occurrence is that the movement of the participants presented as an activation in the primary motor cortex, resulting in a false positive result. However, the region appeared frequently with both datasets suggesting that it deserves further investigation in the future.

Most fMRI studies utilise group level statistics using general linear model approach in order to identify significant brain regions that could be affected. This method operates with p-values as a statistical test to indicate relevant ROIs showing different activities between groups or conditions. These statistics are also corrected with FWR FDR corrections. Machine learning methods on the other hand aim to classify each subject into one of the groups or conditions, and the overall classification accuracy is used to measure success. The significant variables in the general linear model approach does not necessarily mean high classification accuracy^54–56^. Furthermore, our method only uses the preprocessed data which doesn’t contain any correction making it more robust for the classification task.

One limitation of this study is the low number of participants (n:78) which is a frequent problem in most fMRI studies since the recruitment of the participants is difficult. However, we mean to overcome this by utilising the ADNI database. We tested the algorithms with two unrelated datasets and obtained consistent and accurate results, validating our approach. While, in our case the low sensitivity of some models may be attributed to the small sample size, the tests on the ADNI dataset showed higher specificity. Nevertheless, there were still some misclassifications, possibly due to the heterogeneity of the patient population in both datasets. Neurodegenerative processes can affect different parts of the brain for different individuals so it is hard to build a model that can reliably detect MCI as a general condition. Also, MCI is not limited to progressing only to AD but can also develop into other types of dementia which increase the heterogeneity of the group. While the patient heterogeneity is an important limitation, signalling MCI could be beneficial independently from the exact pathological cause since it provides an optimal therapeutic window for drug and lifestyle interventions in all pathologic conditions. Despite these reasons, multiple feature selection and machine learning algorithms were able to perform well on two unrelated datasets which increase the validity of our findings. Another limitation is that we used cross-validation to measure the performance of these models which can increase the models’ performances since we used it in both the feature selection and classification phases. However, it is essential to clarify that our training and testing sets were well-defined, and we tried to minimize data leakage during the cross-validation process. When evaluating various feature subsets, we adhered to a robust cross-validation approach, ensuring that the test sets remained unseen by the models. A notable study by Wen et al. highlighted the significance of addressing data leakage issues, particularly the absence of an independent test set^57^. This concern is relevant to our study, as the use of cross-validation in feature selection may prioritize features optimizing performance within the utilized dataset rather than those that generalize well across different datasets. Despite this, an observation in our study is the presence of multiple overlapping features among the most frequently occurring ones in the two datasets, providing some validation to our findings Also our goal was to test different methods that could possibly be used to differentiate HC from MCI patients and the same approach was used in other studies as well with limited number of participants^13,21,41^. In further studies we aim to use other approaches combining our and the ADNI database to further increase the robustness of our methods. One possibility is to use the ADNI dataset for model training and our dataset for testing which could potentially eliminate the issue of overfitting. One other possible approach is to concatenate the two datasets and see how the algorithms perform. Precise evaluation of the false positive and false negative classifications could also help determine why the models misclassify certain patients but not others.

In conclusion, we propose a method for classifying MCI and HC groups using three voxel-based metrics on two unrelated datasets. The results highlight the potential application of the three metrics with the above-mentioned feature selection and machine learning algorithms. The selected ROIs show promising results since they coincide with the results of the current research regarding the affected brain regions by AD pathology. However, the method requires further validation as cross-validation may cause overfitting. The involvement of precentral gyrus could be a future research question since it is among the most frequently selected regions in both the ADNI and our local dataset.

## Data Availability

The datasets used and/or analyzed during the current study available from the corresponding author on reasonable request.
